# Microtubule stabilising peptides rescue tau phenotypes in-vivo

**DOI:** 10.1038/srep38224

**Published:** 2016-12-02

**Authors:** Shmma Quraishe, Megan Sealey, Louise Cranfield, Amritpal Mudher

**Affiliations:** 1 Centre for Biological Sciences, Faculty of Natural and Environmental Sciences, Building 85, University of Southampton, Southampton, SO17 1BJ, UK

## Abstract

The microtubule cytoskeleton is a highly dynamic, filamentous network underpinning cellular structure and function. In Alzheimer’s disease, the microtubule cytoskeleton is compromised, leading to neuronal dysfunction and eventually cell death. There are currently no disease-modifying therapies to slow down or halt disease progression. However, microtubule stabilisation is a promising therapeutic strategy that is being explored. We previously investigated the disease-modifying potential of a microtubule-stabilising peptide NAP (NAPVSIPQ) in a well-established *Drosophila* model of tauopathy characterised by microtubule breakdown and axonal transport deficits. NAP prevented as well as reversed these phenotypes even after they had become established. In this study, we investigate the neuroprotective capabilities of an analogous peptide SAL (SALLRSIPA). We found that SAL mimicked NAP’s protective effects, by preventing axonal transport disruption and improving behavioural deficits, suggesting both NAP and SAL may act via a common mechanism. Both peptides contain a putative ‘SIP’ (Ser-Ile-Pro) domain that is important for interactions with microtubule end-binding proteins. Our data suggests this domain may be central to the microtubule stabilising function of both peptides and the mechanism by which they rescue phenotypes in this model of tauopathy. Our observations support microtubule stabilisation as a promising disease-modifying therapeutic strategy for tauopathies like Alzheimer’s disease.

Alzheimer’s disease (AD) is the commonest cause of dementia in the elderly. It is characterised by progressive cognitive decline associated with neuronal dysfunction and death. Extracellular plaques made up of Aβ peptide and intraneuronal filaments/tangles composed of abnormal, highly phosphorylated forms of tau, neuropathologically define AD. Though this disease was first described over 100 years ago, disease-modifying therapies are still elusive and AD is on the rise. It is estimated that 65.7 million people worldwide will be living with dementia by 2030^1^. Clearly, there is an urgent, unmet need for disease-modifying therapies to treat AD^2^,^3^.

Tau is a microtubule binding protein that is important for the assembly, maintenance and stability of microtubules (MT). Hyper-phosphorylation of tau, as found in AD, decreases its affinity for tubulin, compromising its ability to stabilise the MTs and thus disrupting cytoskeletal integrity and axonal transport^4^,^5^,^6^. These phospho-tau mediated phenotypes are evident in many *in-vivo* models of tauopathy^7^,^8^,^9^,^10^ including our own *Drosophila* model in which a wild-type, highly phosphorylated isoform of human tau (htau^0N3R^) is expressed^11^.

*Drosophila* is an established model system for analysing the cellular and molecular mechanisms that underlie a variety of neurodegenerative diseases, particularly tau-associated diseases^12^,^13^. Htau^0N3R^-expression in this model causes neuronal dysfunction, characterised by MT destabilisation^6^, axonal transport disruption^11^, synaptic defects^12^ and behavioural impairments^11^. This model has been used to explore the effectiveness of disease‐modifying interventions to either reduce tau phosphorylation or enhance MT stabilisation^6^,^14^. Treatment with NAP (NAPVSIPQ also known as ‘davunetide’), a small octapeptide derived from activity dependent neuroprotective protein (ADNP)^15^ effectively restores MT integrity^14^,^16^ and protects MT-dependent axonal transport in both rodent^17^ and *Drosophila*^14^ models of disease. Moreover, in the *Drosophila* model, NAP-mediated protection against htau^0N3R^ phenotypes spans cellular and molecular dysfunction through to behavioural defects *in-vivo*^14^.

NAP is reported to modulate MT dynamics in a fashion similar to MT plus-end tracking proteins (+TIPS)^18^. +TIPS target the dynamic ends of MTs, catalysing immediate changes in MT stability, directionality and growth^19^,^20^. However, the majority of +TIPs do not interact directly with the MT plus-end and/or MT lattice. Instead, this interaction occurs through end-binding proteins (EBs), which recognise and bind conserved Ser-x-Ile-Pro (SxIP) polypeptide motifs within +TIPs^21^. In addition to the classical ‘SxIP’ motif, ‘SIP’ and ‘IP’ sequences are also reported to mediate EB interaction with its binding partners^22^. NAP contains a ‘SIP’ motif within its amino acid sequence (NAPV**SIP**Q). It interacts *in-vitro*^18^ with both EB1, a key regulator of MT dynamics and polymerisation^23^,^24^ and EB3 a central component in dendritic spine formation^25^. Silencing of either EB1 or EB3 abolishes NAP’s protective activity in PC12 cells. Furthermore, silencing of EB3 in primary cortical neurons inhibits NAP-mediated dendritic spine formation^18^. A novel NAP analogue, SKIP, is reported to bind NAP and enhance axonal transport in ADNP-deficient mice^26^. It is therefore conceivable that NAP restores MT integrity and function in our *Drosophila* model of tauopathy by interacting with EB’s via its SIP domain. The data presented here tests this hypothesis by exploring the MT stabilising potential of another analogous peptide called SAL (SALLRSIPA also termed ADNF-9), which also contains a SIP domain. SAL is derived from the glial precursor protein, activity dependent neurotrophic factor (ADNF). It exhibits similar neuroprotective capabilities to NAP in numerous animal and cell models of injury and disease^27^,^28^,^29^,^30^,^31^.

In this study we investigated whether SAL, like NAP could also protect against htau^0N3R^-mediated neuronal dysfunction in our *Drosophila* model of tauopathy. The phenotypes that arise in this model occur as a direct or indirect consequence of MT breakdown. This model was therefore ideally suited to test SAL’s ability to modulate MT integrity and thus determine the importance of the SIP domain in MT stabilising therapeutic approaches.

## Results

### SAL prevents htau^0N3R^-mediated locomotor impairment

**Figure 1:**
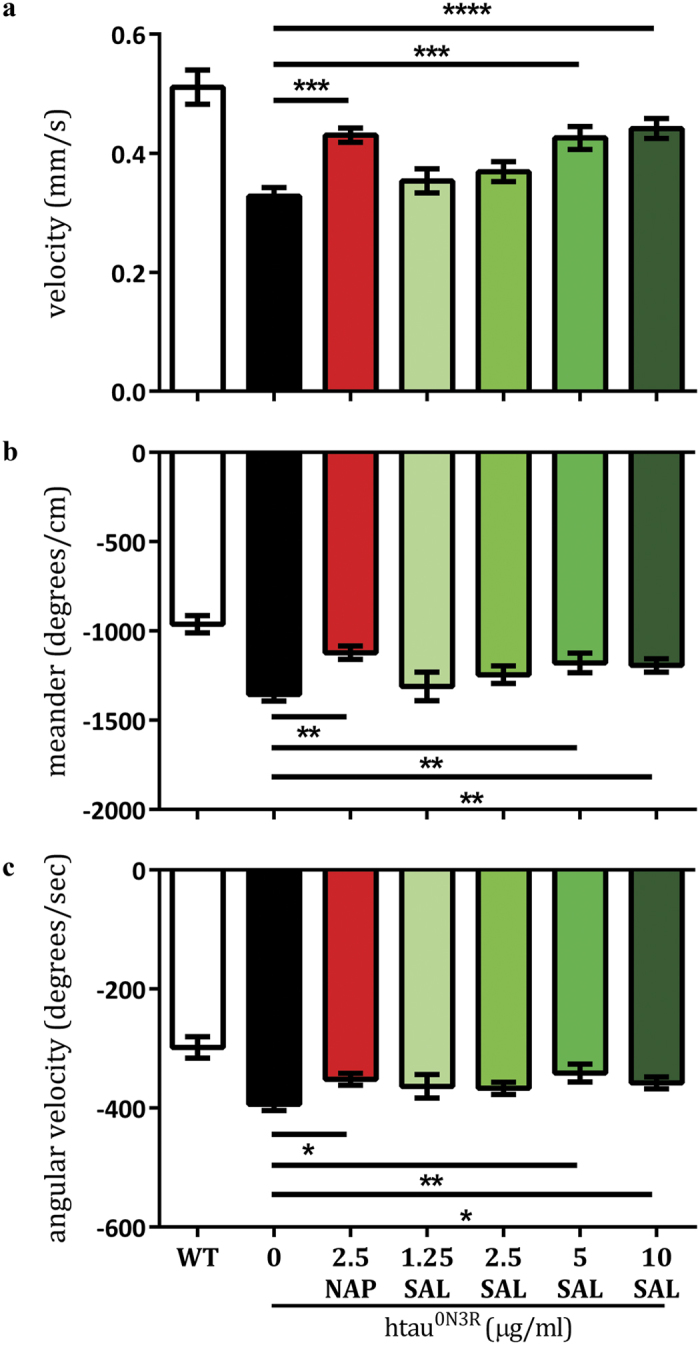
SAL improves the locomotor phenotype in htau^0N3R^-expressing *Drosophila* larvae. Crawling performance (velocity, meander (turning rate per distance travelled) and angular velocity (turning rate per time elapsed)) was quantified using the tracking-software Ethovision. NAP treatment significantly improved all crawling parameters (**a–c**, red bar) compared to untreated htau^0N3R^-expressing larvae (**a–c**, black bar). A dose dependent improvement in all crawling parameters was observed in SAL-treated larvae. 5 μg/ml SAL (green bars) and 10 μg/ml SAL (dark green bars) significantly improved crawling performance: velocity (**a**), meander (**b**) and angular velocity (**c**) compared to untreated htau^0N3R^-expressing larvae (black bars). Data were analysed with an unpaired Students t-test. Error bars represent mean ± S.E.M., *P < 0.05, **P < 0.01, ***P < 0.001, ****P < 0.0001 as determined by unpaired Student’s two-tailed *t*-test, *n* = wt (21–27), htau^0N3R^ (39–44), 2.5 μg/ml NAP (12–14), 1.25 μg/ml SAL (16–17), 2.5 μg/ml SAL (38–40), 5 μg/ml SAL (29–31) and 10 μg/ml SAL (40–49).

Expression of htau^0N3R^ within motor neurons of *Drosophila* manifests in a number of distinct phenotypes including crawling defects in larvae^11^. Larvae expressing htau^0N3R^ exhibit a restricted and non-continuous crawling behaviour indicative of impaired neuronal function^14^,^32^. Using the image-tracking software Ethovision, crawling parameters were quantified including velocity, meander (turning rate per distance travelled) and angular velocity (turning rate per time elapsed). Meander and angular velocity are presented on a negative measurement scale in Ethovision. As previously shown^14^, 2.5 μg/ml NAP treatment significantly improved velocity (Fig. 1a, red bar, *p* = 0.0003, *n* = 14) compared to untreated htau^0N3R^-expressing larvae (Fig. 1a, black bar, *n* = 39). Meander and angular velocity were also significantly improved in NAP-treated larvae (Fig. 1b, red bar, *p* = 0.017, *n* = 12 and Fig. 1c, red bar, *p* = 0.0426, *n* = 12, respectively) compared to untreated htau^0N3R^-expressing larvae (Fig. 1b and c, black bar, *n* = 44 and 39, respectively). Larvae treated with SAL displayed a dose dependent improvement in all parameters tested. Treatment with 1.25 μg/ml and 2.5 μg/ml of SAL (Fig. 1a, pale green bar, *n* = 16 and light green bar, *n* = 39, respectively) did not improve velocity compared to untreated htau^0N3R^-expressing larvae (Fig. 1a, black bar, *n* = 39). However, 5 μg/ml SAL (Fig. 1a, green bar, *p* = 0.0001, *n* = 29) and 10 μg/ml SAL (Fig. 1a, dark green bar, *p* = <0.0001, *n* = 40) significantly improved velocity compared to untreated htau^0N3R^ larvae (Fig. 1a, black bar, *n* = 39). Meander of htau^0N3R^-expressing larvae treated with 1.25 μg/ml and 2.5 μg/ml of SAL (Fig. 1b, pale green bar, *n* = 17 and light green bar, *n* = 40, respectively) did not improve compared to untreated htau^0N3R^-expressing larvae (Fig. 1b, black bar, *n* = 44). However, 5 μg/ml SAL (Fig. 1b, green bar, *p* = 0.0061, *n* = 31) and 10 μg/ml SAL (Fig. 1b, dark green bar, *p* = 0.0022, *n* = 49) significantly improved meander compared to untreated htau^0N3R^ larvae (Fig. 1b, black bar, *n* = 44). Similar to the results for the previous crawling parameters assessed, angular velocity of htau^0N3R^-expressing larvae treated with 1.25 μg/ml and 2.5 μg/ml of SAL (Fig. 1c, pale green bar, *n* = 17 and light green bar, *n* = 38, respectively) did not improve compared to untreated htau^0N3R^-expressing larvae (Fig. 1c, black bar, *n* = 39). However, as also demonstrated for velocity and meander, 5 μg/ml SAL (Fig. 1c, green bar, *p* = 0.0044, *n* = 31) and 10 μg/ml SAL (Fig. 1c, dark green bar, *p* = 0.0165, *n* = 46) significantly improved angular velocity compared to untreated htau^0N3R^ larvae (Fig. 1b, black bar, *n* = 39). Treatment with SAL (2.5 μg/ml and 10 μg/ml) did not alter the crawling performance of controls compared to untreated controls (Supplementary Figure 1b–d). Data were analysed by unpaired Student’s two-tailed t-test.

### SAL prevents htau^0N3R^-mediated disruption of axonal transport

**Figure 2:**
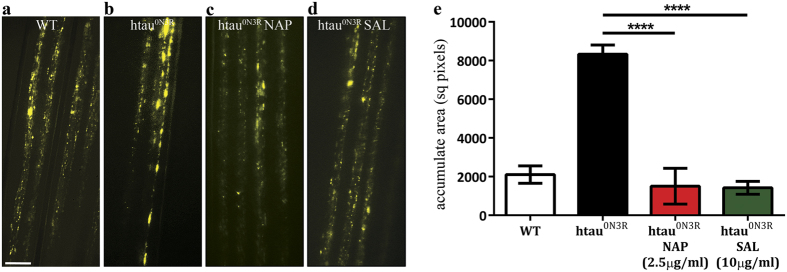
SAL prevents axonal transport deficits in htau^0N3R^-expressing *Drosophila* larvae. Wild-type, control untreated larvae exhibited a homogeneous distribution of vGFP fluorescence in peripheral nerves indicative of an efficient axonal transport system (**a**). Htau^0N3R^ larvae exhibited accumulation of vGFP, indicative of disrupted axonal transport (**b**). 2.5 μg/ml NAP prevented accumulation of vGFP in htau^0N3R^ larval motor neurons (**c**). 10 μg/ml SAL also prevented accumulation of vGFP in htau^0N3R^ larval motor neurons (**d**). The total area of axons (within a defined region) encompassed by vGFP accumulates was greater in htau^0N3R^ larvae compared to controls. 2.5 μg/ml NAP and 10 μg/ml SAL reduced the area covered by vesicular accumulates back to control levels (**e**). Data were analysed by one-way ANOVA with Bonferroni’s correction. Error bars represent mean ± S.E.M., P**** < 0.0001; *n* = wt (10), htau^0N3R^ (9), 2.5 μg/ml NAP (5), 10 μg/ml SAL (9). Scale bar: 10 μm.

In this model, axonal transport is disrupted because of MT breakdown^6^,^11^. This can be visualised *in-vivo* and in real time through the expression of vesicular neuropeptide-Y-GFP (vGFP) in the motor neurons of living intact larvae^11^. Efficient axonal transport in untreated wild-type (wt) control larvae was evident by a homogeneous distribution of vGFP in peripheral nerves (Fig. 2a, n = 10). In htau^0N3R^-expressing larvae, large vGFP accumulates were distributed within the axons of peripheral nerves, illustrating profound axonal transport disruptions (Fig. 2b and e, black bar, *p *< 0.0001, *n* = 9). As previously demonstrated, 2.5 μg/ml of NAP prevented axonal transport deficits in htau^0N3R^-expressing larvae, thus restoring MT integrity (Fig. 2c and e, red bar, *p *< 0.0001, *n* = 5)^14^. Given that SAL improved crawling behaviour at higher doses (5 μg/ml and 10 μg/ml) compared to the more efficacious NAP (2.5 μg/ml), we assessed axonal transport deficits in htau^0N3R^-expressing larvae treated with 10 μg/ml of SAL. We found that 10 μg/ml of SAL prevented axonal transport deficits as effectively as 2.5 μg/ml of NAP (Fig. 2d and e, dark green bar, *p *< 0.0001, *n* = 9). Quantification of the total axonal area occupied by vGFP accumulates confirmed these results (Fig. 2e). Data were analysed by one-way ANOVA with Bonferroni’s correction.

### SAL does not alter tau phosphorylation

**Figure 3:**
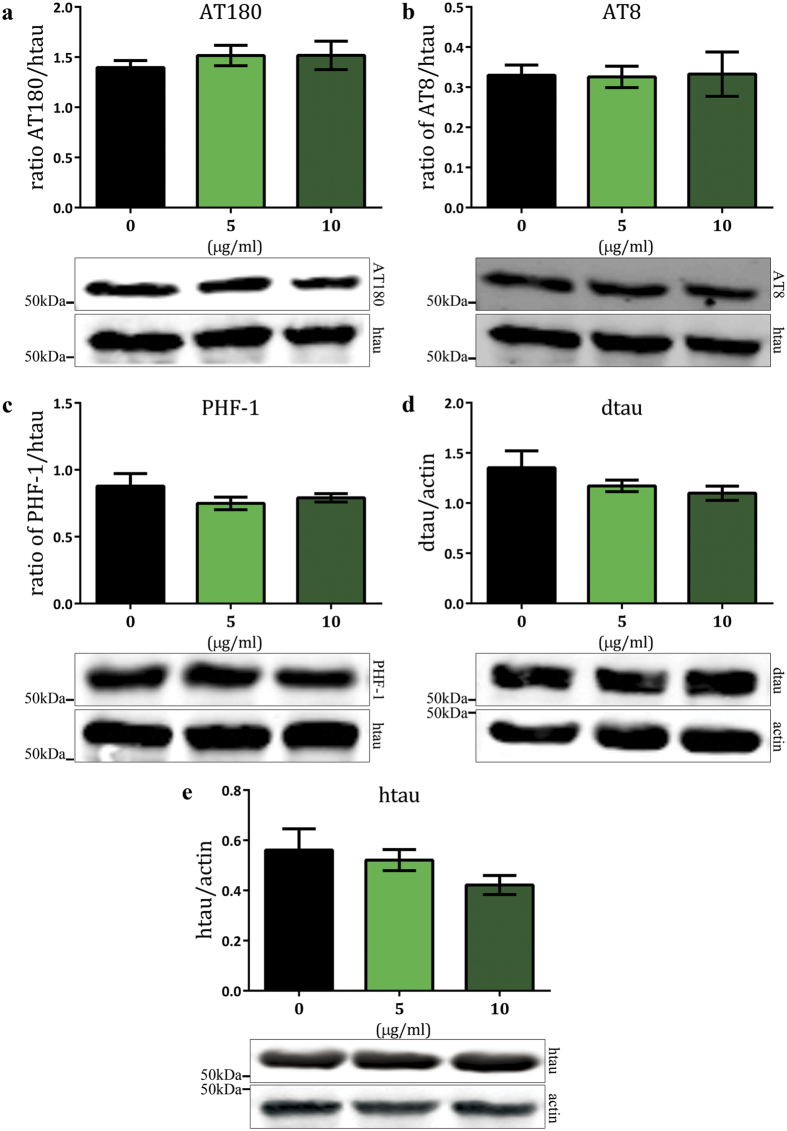
SAL does not alter total htau^0N3R^ levels or phosphorylation at a number of sites relevant to AD. For each phospho-tau antigen, intensity of signal (pixels/mm^2^) was normalised to total tau levels. There was no significant change in the levels of the phospho-tau epitopes detected by AT180 (**a**), AT8 (**b**), and PHF-1 (**c**) after treatment with 5 μg/ml SAL (green bars) and 10 μg/ml SAL (dark green bars) SAL. Total dtau levels were not altered by SAL treatment (**d**). Representative blots are shown (a-d). All lanes were run on the same gel. Data were analysed by one-way ANOVA with Bonferroni’s correction. Error bars represent mean ± S.E.M; *n* = htau^0N3R^ (5), 5 μg/ml SAL (5), 10 μg/ml SAL (5).

The MT binding and stabilising ability of htau is reduced by hyper-phosphorylation^5^,^6^, leading to compromised cytoskeletal integrity. Reduction of tau phosphorylation through genetic and chemical manipulation is known to improve htau^0N3R^-mediated phenotypes^6^,^33^. However, we previously reported that NAP does not alter the phosphorylation status of htau^0N3R^ and was conferring protection by another mechanism^14^. We therefore investigated whether SAL was preventing the emergence of the htau^0N3R^-mediated phenotypes by reducing tau phosphorylation or whether it too, was bypassing the pathogenic phospho-htau^0N3R^ protein. We examined a number of phospho-tau epitopes associated with AD in both SAL-treated and untreated htau^0N3R^-expressing 1–3 day old flies. Each phospho-tau antigen (intensity of signal, pixels/mm^2^) was normalised to total htau levels on the same blot. This enabled assessment of changes in the phosphorylation status of tau, independent of changes in total tau levels. The AT180 monoclonal antibody detects phosphorylation of tau at Thr231. This site is important for MT binding, and phosphorylation, as occurs in AD at this site, inhibits binding of tau to MTs^34^. Htau^0N3R^-expressing flies treated with SAL at 5 or 10 μg/ml did not show changes in tau phosphorylation at the phospho-tau epitope detected by the AT180 antibody compared to untreated htau^0N3R^ larvae (Fig. 3a, *n* = 5). The AT8 monoclonal antibody detects phosphorylation of tau at Ser202/Thr205. Htau^0N3R^-expressing flies treated with SAL at 5 or 10 μg/ml did not show changes in tau phosphorylation at the phospho-tau epitope detected by the AT8 antibody compared to untreated htau^0N3R^ larvae (Fig. 3b, *n* = 5). Another phospho-tau epitope, Ser396/Ser404, that is abnormally phosphorylated in AD can be detected by the PHF-1 antibody^35^. Similar to AT180 and AT8, htau^0N3R^-expressing flies treated with SAL at 5 or 10 μg/ml did not show changes in tau phosphorylation at the phospho-tau epitope detected by the PHF-1 antibody compared to untreated htau^0N3R^ larvae (Fig. 3c, *n* = 5). Total dtau and htau levels were not significantly different in SAL-treated compared to untreated htau^0N3R^-expressing flies (Fig. 3d and e). Actin was used as an additional protein loading control for all samples (Fig. 3d,e and Supplementary Figure 2). We did not include wt control animals in this analysis as they do not express htau^0N3R^ (Supplementary Figure 1a). Wide view panels of all the blots are presented in Supplementary Figure 2. Data were analysed by one-way ANOVA with Bonferroni’s correction.

## Discussion

In this study, we demonstrate that the neuroprotective peptide SAL (SALLRSIPA) provides protection against htau^0N3R^-mediated phenotypes as also demonstrated for an analogous neuroprotective peptide NAP (NAPVSIPQ), albeit at higher doses (5 and 10 μg/ml for SAL compared to 2.5 μg/ml for NAP). SAL significantly improved htau^0N3R^-mediated phenotypes *in-vivo*, including axonal transport disruption and behavioural defects. SAL was able to rescue htau^0N3R^ phenotypes without altering phosphorylation at key disease-associated epitopes. These results are reminiscent of those observed with NAP-treatment in this model of tauopathy^14^. SAL is reported to exhibit a similar neuroprotective profile compared to NAP^36^,^37^. However, several studies have also shown that NAP is more efficacious than SAL^15^,^29^. This is consistent with our observations in the present study.

### SAL and NAP – two neuroprotective peptides

SAL and NAP are short peptides derived from two secreted astroglial parent proteins, ADNF and ADNP (respectively)^15^,^38^,^39^. NAP and SAL were identified as the essential regions of their respective parent proteins for conferring neuroprotection^39^. In subsequent studies, SAL and NAP were found to protect against a variety of cellular insults including neurotoxic drugs such as NMDA^40^ and ethanol^28^,^41^. They were also found to be protective in models of Alzheimer’s disease^7^,^27^,^42^,^43^, diabetic neuropathy^44^, amylolateral sclerosis and ADNP induced tauopathy^10^,^17^,^45^. The molecular mechanism underpinning NAP’s neuroprotective ability is thought to occur by MT stabilisation^46^, which counters axonal transport defects^14^,^17^. It is conceivable that like NAP, SAL also has MT stabilising effects as it has been shown to displace NAP in an *in-vitro* MT binding assay^47^. SAL also promotes neurite outgrowth in rat hippocampal cultures, a function reliant on MT stabilisation and plasticity^48^. The data presented herein supports this further by showing that like NAP, SAL also protects against htau^0N3R^-mediated behavioral and axonal transport defects, which arise due to cytoskeletal destabilisation^6^,^14^. These htau^0N3R^-phenotypes could be attributed to neurodevelopmental effects given the drivers used in this study (D42- and Elav-Gal4) are not exclusively post-mitotic and motor-neuron specific^49^,^50^. D42-Gal4 is expressed in all post-mitotic motor neurons and in some sensory neurons in the peripheral nervous system^49^. Elav-Gal4 is also expressed in post-mitotic neurons, but is transiently expressed in embryonic glial cells and neuroblasts^50^. Importantly, expression of htau^0N3R^ with these drivers results in robust phenotypes, including MT destabilisation and disrupted axonal transport within the motor neurons of *Drosophila*. We have previously shown rescue of htau^0N3R^-mediated axonal transport deficits after just 24 hours NAP treatment^14^. This implies that the protective effects of NAP and SAL target htau^0N3R^-phenotypes that arise in this model due to MT-associated neuronal dysfunction^11^. We previously demonstrated by EM that cytoskeletal integrity in wt (OreR) controls was unaffected by NAP treatment^14^. Unsurprisingly, no effect of NAP was evident in axonal transport or behavioural assays in these wt controls. In contrast, the same ultrastructural analysis showed a disrupted cytoskeleton, which was restored by NAP-treatment in htau^0N3R^-expressing animals^14^. Likewise, in the current study, wt controls treated with SAL did not show any statistically significant differences in locomotor performance compared to untreated controls, as illustrated in Supplementary Figure 1b–d. An important point to note is that wt strains such as OreR are more robust when compared to isogenised, Gal4/UAS strains which may be susceptible to Gal4 titrations. It would have been ideal to confirm the tau-specific, neuroprotective effect of these peptides, by assessing their impact on transgenic control lines with a UAS background (e.g. UAS-LacZ). However, our primary aim in this study was to determine if SAL-treatment significantly affected htau^0N3R^-phenotypes arising because of MT destabilisation. As such, treated and untreated *Drosophila* expressing htau^0N3R^ were reared and tested alongside each other to minimise any titration and genetic artefacts. Interestingly, we found that axonal transport deficits could be prevented and efficient axonal transport maintained in htau^0N3R^-expressing animals at a comparable level to robust wt OreR control larvae.

### Mode of action of SAL and NAP

Hyper-phosphorylated tau is considered to be the toxic species in tauopathies. It is believed to cause degeneration both due to loss of MT-binding function and accumulation of toxic tau aggregates^51^,^52^. Tau-centric disease-modifying strategies rescue tau phenotypes by reducing tau phosphorylation^11^,^53^, increasing MT stabilisation^9^,^14^,^46^,^54^ or reducing tau aggregation^55^. Our data imply neuroprotective effects independent of reductions in tau phosphorylation, but whether these peptides impact on tau aggregation remains to be determined. Interestingly, we have previously shown that inhibition of GSK-3β rescues tau phenotypes and restores MT integrity, by reducing tau phosphorylation with a consequent increase in tau protein levels and insoluble granular tau oligomers^56^. In the present study, we did not observe any significant changes in total htau^0N3R^ protein and phosphorylation levels after treatment with SAL and as demonstrated for NAP^14^. Previous findings from our lab, as well as other studies conducted in rodent and cell culture models of tauopathy, strongly suggest that NAP neuroprotects by fortification of MTs^14^,^16^,^17^,^18^,^57^.

In this study, we assessed the neuroprotective capabilities of both NAP and SAL peptides individually. SAL showed dose-dependent neuroprotective effects, consistent with our previous observations for NAP^14^. Other studies have also reported dose-dependent neuroprotective effects for both peptides^29^. In a rat model of cholinotoxicity, NAP was more efficacious compared to SAL in cholinergic protection^29^. NAP has also been shown to be more effective then SAL in providing long term protection against loss of spatial memory in apolipoprotein E-deficient mice^15^ and AF64A-treated animals^29^. A combinatorial peptide approach would also have been interesting but is beyond the scope of the current study. However, previous studies have investigated the protective effects of combining both NAP and SAL. A few studies have shown that both peptides are more efficacious together, than either alone^58^,^59^,^60^,^61^. These peptides do not exhibit stereo-selectivity^59^. The more stable, all D-amino acid SAL (D-SAL) showed efficacy *in-vivo* and *in-vitro* models of disease^30^,^31^. In a model of fetal alcohol syndrome (FAS), administration of both D-NAP and D-SAL reduced fetal demise, however, no significant differences between combination and individual drug treatments were seen. In the same study, apolipoprotein E knockout mice treated with both D-NAP and D-SAL showed improved performance in the Morris water maze^59^. In another study, NAP alone was effective in preventing alcohol-induced fetal death, whereas SAL at the same dose was not protective. However, a combinatorial treatment with NAP and SAL was more effective in preventing growth restriction due to prenatal alcohol treatment^60^. These studies suggest a dose-dependent, synergistic effect rather than an additive effect. The differences reported in the literature and the differences in efficacious dose that we too observe may be attributable to the non-homologous amino acids either side of the SIP motif in the two peptides.

Interestingly, both peptides are derived from parent proteins that are secreted by glial cells in response to vasoactive intestinal peptide (VIP)^15^,^38^. VIP is expressed under conditions of stress and one of the early events that occurs during stress or insult mediated injury is a dynamic reorganisation of the cytoskeleton^62^. Both NAP and SAL (NAPVSIPQ and SALLRSIPA) contain a SIP motif^63^. The ‘SIP’ motif within NAP has been implicated in protection against ethanol and tetrodotoxin toxicity in cortical neurons^40^. Substitution of proline (P) with alanine (A) abolishes neuroprotection against oxidative stress (H_2_O_2_) in pheochromocytoma (PC12) cells^64^. Indeed, the SIP motif of NAP and SAL is essential for neuroprotection and interaction with key MT end-binding proteins EB1 and EB3, promoting MT assembly and neuronal plasticity^18^,^26^. Collectively, our data and the studies discussed imply that SAL acts in a similar manner as NAP to confer neuroprotection. However, since the molecular mode of action of SAL has not been explored as comprehensively as that of NAP, this cannot be concluded unequivocally without further investigations.

The data presented here supports the use of SIP containing neuropeptides like NAP and SAL for protection against MT destabilisation such as that seen in tauopathies. Importantly, this work highlights MT stabilisation as a disease-modifying therapeutic strategy that holds great promise for tauopathies like AD where abnormal tau-mediated MT dysfunction is evident.

## Methods

### *Drosophila* genotypes and drug treatments

Transgenic expression of htau^0N3R^ (y^1^w^1118^; P{UAS-MAPT.A}59 A: Bloomington Stock Centre, stock no. 181) was directed to *Drosophila melanogaster* motor neurons using either D42-Gal4, or the D42-Gal4 driver fused to vesicular GFP-tagged neuropeptide-Y (D42-GAL4.UAS-NPY:GFP) as previously described^11^. Pan-neural expression was established with the Elav-Gal4 driver. Female virgin flies homozygous for the D42 or Elav driver were crossed to male flies homozygous for htau^0N3R^ under the UAS promoter (+;+; UAS-htau^0N3R^), or with Oregon-R (OreR) wt, control males. Stocks and transgenic crosses were maintained at 23 °C on a 12 h light/dark cycle. Flies were raised on basic food consisting of malt extract, maize meal, soya flour, agar, granulated sugar, yeast, and propionic acid. NAP (NAPVSIPQ) and SAL (SALLRSIPA) (*L-*isoforms synthesised by Peptide Protein Research Ltd, UK) were delivered to basic fly food at a final concentration of 2.5 μg/ml (NAP) and 1.25 μg/ml, 2.5 μg/ml, 5 μg/ml or 10 μg/ml (SAL). Late L3-stage larvae were selected for by size and wandering behaviour.

### Larval locomotion assay

Larval locomotion analysis was conducted using a semi-quantitative assay as previously described^32^. Briefly, crawling behaviour was analysed on 1% agarose plates dyed with 0.1% w/v Alcian blue (Hopkin and Williams, UK). L3 larvae were positioned in the centre of each plate and allowed to acclimatise for 2 min prior to testing. Open field activity was recorded for 2 min (trial 1). This was further repeated for 2 more trials. Wherever possible, genotypes and treatments were randomised between adjacent plates. Videos of larval locomotion were analysed in Ethovision 3.0 software (Noldus) to determine velocity, angular velocity and meander.

### *In-vivo* axonal transport analysis

All treatment groups were subjected to a 3–4 hour timed lay on apple juice agar plates. F1 eggs were transferred to either basic, NAP or SAL treated food. Larvae were left to develop to L3 wandering stage (day 5). Axonal transport analysis was conducted as previously described^11^,^65^. Briefly, L3 larvae were anaesthetised in diethylether vapour for 15 min, immobilised on glass slides in 1% agarose ventral face up and mounted under coverslips. Peripheral nerves were analysed between the 2^nd^ and 4^th^ denticle bands. For total area acquisition, vGFP accumulates were imaged at x63 on an Axioplan2 Epifluorescence Microscope (Zeiss), and thresholded in Metamorph software (Molecular Devices, CA, USA).

### Western blotting

1–3 day old adult fly heads were homogenised in buffer (150 mM NaCl, 50 mM MES pH6.8, 1% triton-X, protease inhibitor cocktail and 1% SDS). For phospho-tau epitope detection, the following cocktail of phosphatase and kinase inhibitors was also added: 30 mM sodium fluoride, 20 mM sodium pyrophosphate, 40 mM 2-glycerophosphate, 3.5 mM sodium orthovanadate and 10 μM staurosporin. Samples were spun for 2 mins at 3,000 g; the supernatant was removed and heated for 5 mins at 95 °C in Laemmli buffer. Samples were subjected to standard 10% SDS-PAGE and transferred to Protran Nitrocellulose Membrane (Whatman, UK). Blots were probed with the following primary antibodies: anti-human tau (1:15,000, Dako, UK), anti-dtau (1:500, gifted by Prof. St. Johnston, University of Cambridge, UK), anti-actin (1:5000, Abcam, UK) and anti-phospho-tau: PHF-1 (1:2000, gifted by Dr. Peter Davies, Albert Einstein College of Medicine; Bronx, NY), AT180 (1:100, Source, Biosciences, UK), AT8 (1:800, Source, Biosciences, UK). Signal was detected using fluorescently-conjugated secondary antibodies; goat anti-mouse (Alexa-Fluor, Invitrogen, UK) and goat anti-rabbit (IRDye, Licor, UK), used at 1:20,000, and quantified with an Odyssey Infrared Imaging Scanner (LiCor) at 700 nm and 800 nm to give intensity values in pixels/mm^2^.

### Statistics

Statistical analysis was carried out in Prism 5.0 (GraphPad, University of Southampton, Southampton, UK), using a *T*-test or one-way ANOVA with a *post hoc* Bonferroni’s Multiple Comparison Test. All values are reported as means ± S.E.M. *P *< 0.05 was considered significant.

## Additional Information

**How to cite this article**: Quraishe, S. *et al*. Microtubule stabilising peptides rescue tau phenotypes in-vivo. *Sci. Rep.* **6**, 38224; doi: 10.1038/srep38224 (2016).

**Publisher's note:** Springer Nature remains neutral with regard to jurisdictional claims in published maps and institutional affiliations.

## Supplementary Material

Supplementary Figures
