# Corrigendum: Microtubule stabilising peptides rescue tau phenotypes in-vivo

**DOI:** 10.1038/srep40833

**Published:** 2017-02-01

**Authors:** Shmma Quraishe, Megan Sealey, Louise Cranfield, Amritpal Mudher

Scientific Reports 6: Article number: 38224; 10.1038/srep38224 published online: 12 02 2016; updated: 02 01 2017.

The Acknowledgements section in this Article is incomplete.

“This work was supported by the Wessex Medical Trust, the Alzheimer’s Society and the Henry Smith’s foundation. Thanks to Prof. St. Johnstone (University of Cambridge, UK) for providing the dtau antibody and Dr. Peter Davies (Albert Einstein College of Medicine; Bronx, NY) for providing the PHF-1 antibody”.

should read:

“This work was supported by the Wessex Medical Trust, the Alzheimer’s Society and the Henry Smith’s foundation. Thanks to Prof. St. Johnstone (University of Cambridge, UK) for providing the dtau antibody and Dr. Peter Davies (Albert Einstein College of Medicine; Bronx, NY) for providing the PHF-1 antibody. Thanks to the ARUK South Coast Network for contributing to publication costs”.

