# Supplementary material for: Microtubule stabilising peptides rescue tau phenotypes in-vivo
Source: Sci Rep. 2016 Dec 2;6:38224. doi: 10.1038/srep38224 (PMC5133624; doi:10.1038/srep38224)
Supplement: Supplementary Figures [file srep38224-s1.pdf]

Microtubule stabilising peptides rescue tau phenotypes *in-vivo*

Shmma Quraishe*[(a)], Megan Sealey[(a)], Louise Cranfield[(a)], Amritpal Mudher[(a)]

Supplementary Figures

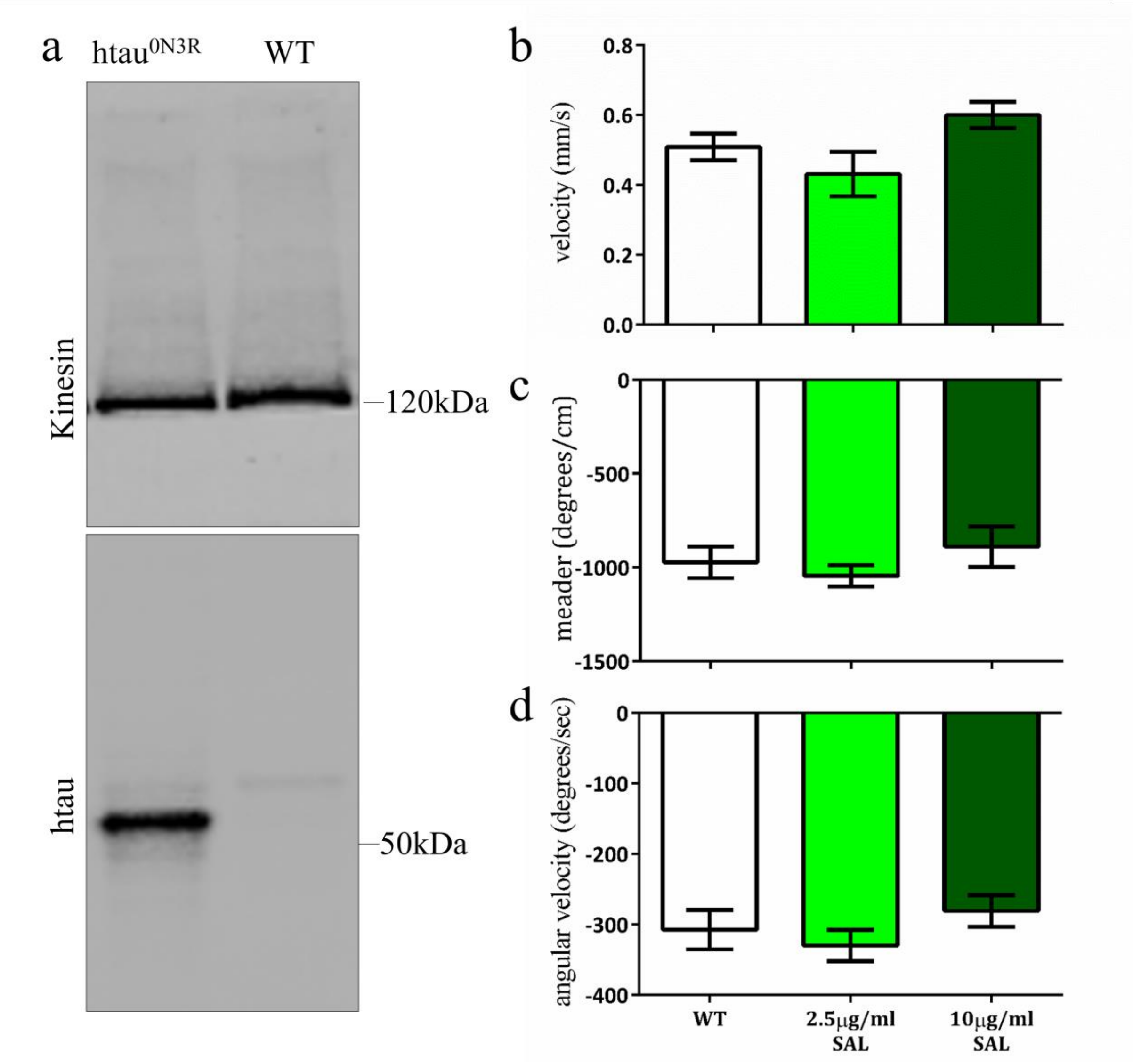

Supplementary Figure 1. WT *Drosophila* do not express htau and SAL does not affect crawling behaviour of controls. Expression of htau was assessed in transgenic (htau^{0N3R}-expressing) compared to wt *Drosophila*. As expected, the htau band at 55kDa was only present in transgenic animals and not in wt animals. Kinesin was used as a loading control. Representative blots are shown (a). Crawling performance of wt controls was quantified using the tracking-software Ethovision. Treatment with 2.5μg/ml SAL (light green bars) and

10µg/ml SAL (dark green bars) did not alter the crawling performance compared to un-treated wt controls, as assessed by three different measures: velocity (b) meander (c) and angular velocity (d). Error bars represent mean ± S.E.M., $P < 0.05$, data were analysed with an unpaired Students t-test, n = WT (12-13), 2.5µg/ml SAL (13-14), and 10µg/ml SAL (15-17).

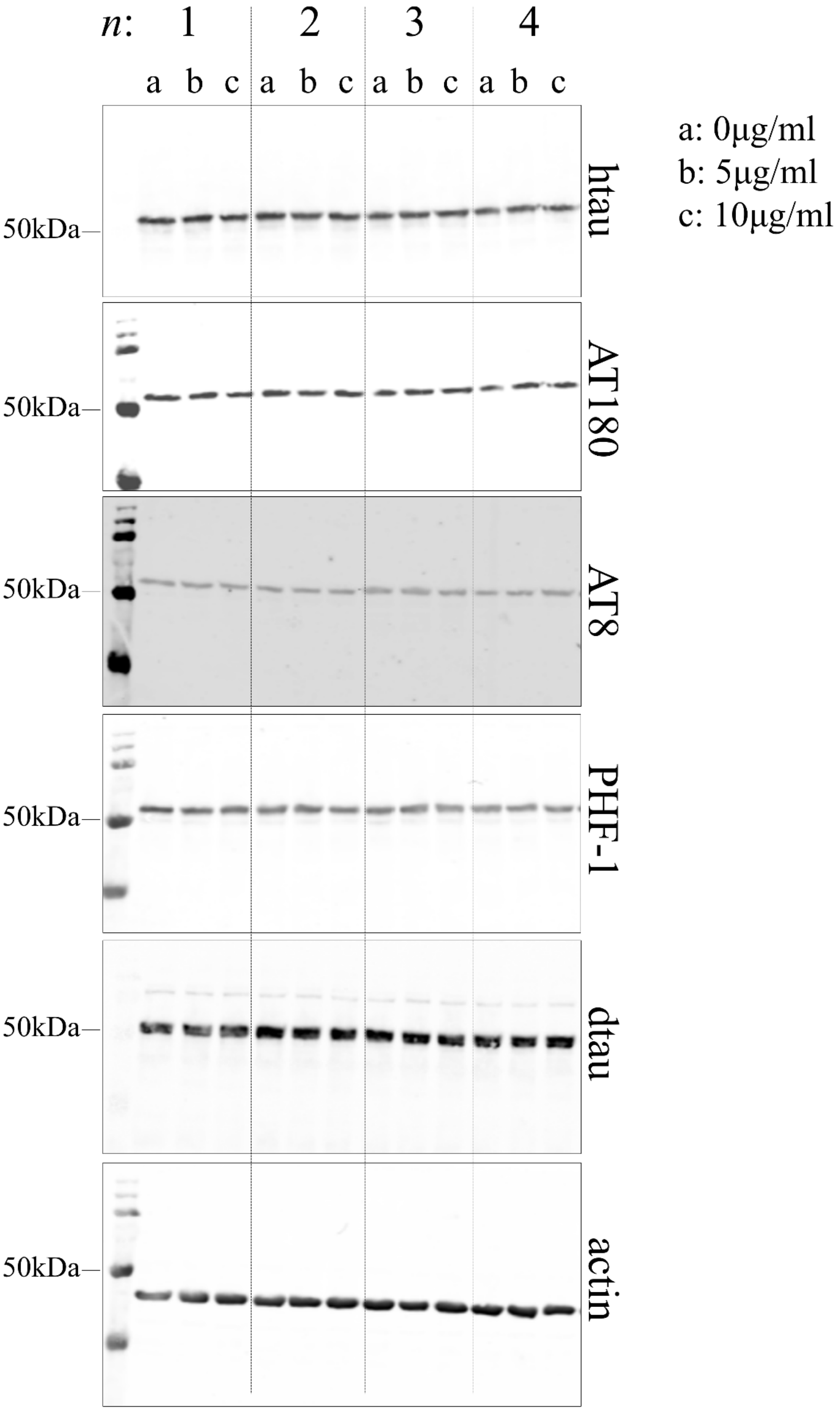

Supplementary Figure 2. SAL does not alter total tau levels or tau phosphorylation at a number of sites relevant to AD. Uncropped blots showing *n* = 4 for htau^{0N3R}-expressing flies treated with a: 0µg/ml SAL, b: 5µg/ml SAL and c: 10µg/ml. Each sample was probed for

htau, AT180, AT8, PHF-1, dtau and actin. Blots were scanned and quantified with an Odyssey Infrared Imaging Scanner (LiCor) at 700nm and 800nm to give intensity values in pixels/mm^2, which were used for analysis. Only one distinct band was present at the correct molecular weight for each antibody (cropped bands are shown in Figure 3).
